# microRNA-99a inhibiting cell proliferation, migration and invasion by targeting fibroblast growth factor receptor 3 in bladder cancer

**DOI:** 10.3892/ol.2014.1875

**Published:** 2014-02-12

**Authors:** DEYAO WU, YUNFENG ZHOU, HUIXING PAN, JIAN ZHOU, YUANFENG FAN, PING QU

**Affiliations:** Department of Urology, The Fourth Affiliated Hospital of Nantong Medical College, Yancheng City No. 1 People’s Hospital, Yancheng, Jiangsu 224001, P.R. China

**Keywords:** bladder cancer, fibroblast growth factor receptor 3, miR-99a, microRNA

## Abstract

The expression of microRNA-99a (miRNA-99a) has been investigated in a number of human cancers. It has been reported to be downregulated in several types of cancer, including ovarian carcinoma, squamous cell carcinoma of the tongue, squamous cell lung carcinoma, hepatocellular carcinoma, bladder cancer, prostate cancer and childhood adrenocortical tumors. In the present study, the effects of miRNA-99a on bladder cancer cell proliferation, migration and invasion were examined. Following transfection of miRNA-99a, cell viability, cell migration assay, cell invasion, western blot analysis and luciferase assays were conducted in bladder cancer cell lines. It was found that miRNA-99a inhibits cell proliferation, migration and invasion in T24 and EJ cells. Additionally, this study provided the first evidence that miRNA-99a is likely to directly target fibroblast growth factor receptor 3 in bladder cancer. The study provided evidence that miRNA-99a suppresses cell proliferation, migration and invasion by targeting growth factor receptor 3 in bladder cancer cell lines. These results indicated that it could be investigated as a target for therapeutic drugs designed to treat bladder cancer.

## Introduction

Bladder cancer is the most common malignancy of the urinary tract system and represents a significant cause of morbidity and mortality (1). In the United States it was estimated that there would be 70,530 novel cases and 14,680 mortalities due to bladder cancer in 2010 (2). In total, 90% of bladder cancers are urothelial carcinomas, previously known as transitional cell carcinomas, followed by squamous cell carcinoma (5%) and adenocarcinoma (2%). There are two principal forms of bladder cancer: Low-grade superficial tumors and high-grade invasive tumors. In total, ~70% of patients present with non-muscle invasive (superficial) tumors, while the remaining 30% present with muscle-invasive tumors (3). Currently, the main therapeutic method for bladder cancer without metastasis is surgery followed by postoperative intravesical instillation. However, >30% of patients either fail to respond to treatment or suffer recurrent disease within five years, and 50% of patients die from metastatic disease (4). Thus, novel treatments are required to improve the prognosis for patients with bladder cancer.

MicroRNAs (miRNAs), the endogenous small (~22 nt) non-coding RNA molecules, are generally regarded as negative regulators of gene expression that inhibit translation of mRNA by binding to the 3′ untranslated region (3′UTR) of target mRNAs (5). To date, >400 human miRNAs have been identified, while >1,000 miRNAs have been postulated to exist (6). Dysregulation of miRNAs has been implicated in a number of diseases, prominently including cancer, where there are significant differences in miRNA expression profiles of cancer versus normal cells from the tissues of origin (7). Increasingly more evidence revealed that miRNAs are likely to play significant roles in various biological processes, including cell proliferation, apoptosis and tumorigenesis of cancer (8). Accumulated studies have shown that certain miRNAs are overexpressed and some are downregulated in several cancers compared with the normal tissues of origin, indicating that these miRNAs may function as oncogenes or tumor suppressors in the tumorigenesis of various human cancers (9,10). Upregulated miRNAs in cancer may function as oncogenes by negatively regulating tumor suppressors. By contrast, downregulated miRNAs may normally function as tumor suppressor genes and inhibit cancer by regulating oncogenes. Thus, the identification of the target of miRNAs is critical for understanding the function of miRNAs in cancer development and progression. It is also indicated that miRNA may be a target for cancer therapy.

The expression of miR-99a has been investigated in a number of human cancers. It has been reported to be downregulated in several types of cancer, including ovarian carcinoma, squamous cell carcinoma of the tongue, squamous cell lung carcinoma, hepatocellular carcinoma (HCC), bladder cancer, prostate cancer and childhood adrenocortical tumors (11). These findings indicate that miR-99a is widely downregulated in human cancers, suggesting a potential role of miR-99a as a tumor suppressor. However, to date, there are no studies of miR-99a in bladder cancer, thus, we focused on this.

## Materials and methods

### Cells and culture conditions

The human bladder cancer cell lines, T24 and EJ, were obtained from the Institute of Biochemistry and Cell Biology, Shanghai Institutes for Biological Sciences, Chinese Academy of Sciences (Shanghai, China). T24 and EJ cells were cultured in RPMI-1640 medium supplemented with 10% heat-inactivated fetal bovine serum (FBS) (Gibco, Grand Island, NY, USA) under a humidified air atmosphere of 5% CO_2_ at 37°C. The cells were subcultured every two days using trypsin/ethylenediaminetetraacetic acid (EDTA) solution [saline containing 0.05% trypsin, 0.01 M sodium phosphate and 0.53 μM EDTA, (pH 7.4) (Beyotime, Haimen, China)].

### Transfection of miR-99a mimics, scrambled control (NC) and luciferase reporter plasmid

Mature miR-99a mimics and NC were designed and synthesized by GenePharma (Shanghai, China). The insertion fragment was confirmed by DNA sequencing, and cell transfection and cotransfection were performed using Lipofectamine 2000 (Invitrogen Life Technologies, Carlsbad, CA, USA ) according to the manufacturer’s instructions.

### Cell growth/cell viability assay

Cell proliferation was determined by the 3-(4, 5-dimethyl-2-thiazoyl)-2, 5-diphenyl-2H-tetrazolium bromide (MTT; Sigma-Aldrich, Seezle, Germany) assay. The cells were transfected with miR-99a mimics or NC and were seeded in 96-well plates at a density of 3,000 cells per well. At various time points following the treatment, the medium was removed and 20 μl MTT was added to each well. The 96-well plates were incubated at 37°C for 4 h. The plates were centrifuged and the formazan precipitates were dissolved in 200 μl dimethyl sulfoxide (Sigma-Aldrich). The absorbance of the solution was measured at 490 nm using an ELISA reader (Bio-Rad, Richmond, CA, USA). There were six wells for replication for every time point in each group. Proliferation curves were drawn on the basis of the mean absorbance at each time point, and all the experiments were performed in triplicate. The suppression rate was calculated using the following formula: $\text{Suppression rate}=(1-{\text{OD}}_{\text{miR}-99\text{a}}/{\text{OD}}_{\text{miR}-\text{NC}})\times 100$.

### Cell migration and invasion assay

The cell migration and invasion were assayed using a 8 μm-pore polycarbonate membrane Boyden chamber insert in a Transwell apparatus (Costar, Cambridge, MA), with and without Matrigel (BD Biosciences, San Jose, CA). For the invasion assay, a Transwell chamber was placed into a 24-well plate, coated with 30 μl Matrigel and incubated for 40 min at 37°C. The transfected cells (miR-99a mimics and NC) growing in the log phase were treated with trypsin and re-suspended as single-cell solutions. A total of 1×10^5^ cells per well were cultured in RPMI-1640 medium with 2% FBS serum, while 600 μl RPMI-1640 containing 20% FBS was added to the lower chamber. Subsequent to the cells being incubated for 12–24 h at 37°C in a 5% CO_2_ incubator, the migrated cells were fixed with 100% methanol for 2 min, stained in 0.5% crystal violet for 2 min, rinsed in phosphate-buffered saline and then subjected to microscopic inspection (magnification, ×200). The values for invasion and migration were obtained by counting five fields per membrane and represent the average of three independent experiments.

### Western blot analysis

Primary antibodies used in the present study, including FGFR3 (rabbit, polyclonal) and β-actin (rabbit, monoclonal) were products of Bioworld Technology (Louis Park, MN, USA). Equal amounts of the proteins were separated by 10% SDS-PAGE (Beyotime) and transferred to polyvinylidene difluoride membranes (Beyotime). The membranes were then blocked with 5% skimmed milk and incubated overnight with primary antibodies at dilutions specified by the manufacturer’s instructions. Next, the membranes were washed and incubated with the corresponding horseradish peroxidase-conjugated secondary antibody (goat anti-rabbit) at 1:1,000 dilution in tris-buffered saline with Tween (Beyotime). The blot was developed with enhanced chemilluminescence solution (Pierce, Rockford, IL, USA) and photographed by FluorChem imaging system (Alpha Innotech Corp., San Leandro, CA, USA). The intensity of each spot was read and analyzed with AlphaEaseFC software (Alpha Innotech Corp.). β-actin was used as a loading control.

### Luciferase assay

TargetScan 5.2 (http://www.targetscan.org/) and PicTar (http://pictar.mdc-berlin.de/) in order to assess the complementarity of miR-99a to the FGFR3 3′-UTR. Luciferase reporter assays were performed in order to evaluate whether FGFR3 is a successful target for miR-99a. The cells were plated in a 12-well plate at ~90% confluence and transfected with 0.5 μg reporter plasmid, 40 nmol miR-99a mimics or their negative control by Lipofectamine 2000. The primers used for cloning FGFR3 mRNA 3′UTR were as follows: Forward, GGGCTCGAGGGCCACTGGTCCCCAACAATGTG, and reverse, GGGCGGCCGCCCAGTAACAGTACAGAACGA ACCAAC. Each sample was also cotransfected with 0.05 μg pRL-CMV plasmid expressing Renilla Luciferase (Promega, Manheim, Germany) as an internal control for the transfection efficiency. Subsequent to 48 h of transfection, the cells were harvested and lysed, and the luciferase reporter activities were measured using a luminometer (Tecan, Theale, UK). The firefly and renilla luciferase activities were measured with a Infinite^®^ M1000 PRO Luminometer (Tecan, Theale, UK). The firefly luciferase activity was normalized to the renilla luciferase activity for each transfected well. All the experiments were performed in triplicate.

### Statistical analysis

Data were presented as the mean ± standard deviation, and compared using Student’s t-test in Stata 10.0 (College Station, TX, USA). A double-tailed P-value of <0.05 was considered to indicate a statistically significant difference.

## Results

### miR-99a suppresses cell proliferation in bladder cancer T24 and EJ cells

**Figure 1:**
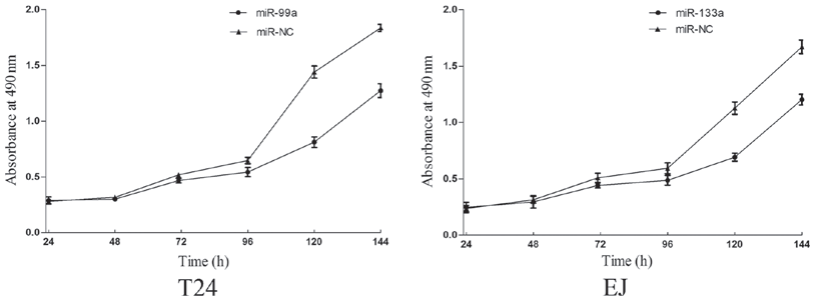
Viability of T24 and EJ cells following transfection of miR-99a. The proliferation of cells was determined by the 3-(4, 5-dimethyl-2-thiazoyl)-2, 5-diphenyl-2H-tetrazolium bromide assay. The results indicated that upregulation of miR-99a significantly suppressed cell proliferation in the bladder cancer cell lines. miR-99a, microRNA-99a; NC, scrambled control. Student’s t test; P<0.05.

In order to investigate the influence of miR-99a on cell proliferation, an MTT method was used. As expected, upregulation of miR-99a significantly inhibited cell proliferation (Fig. 1). The MTT assays revealed that subsequent to 144 h of treatment, the suppression rate of miR-99a reached 34.31±4.5% in T24 cells and 28.01±4.1% in EJ cells. The results indicated that miR-99a may play a significant role in bladder cancer T24 and EJ cells.

### miR-99a suppresses cell migration and invasion in bladder cancer T24 and EJ cells

**Figure 2:**
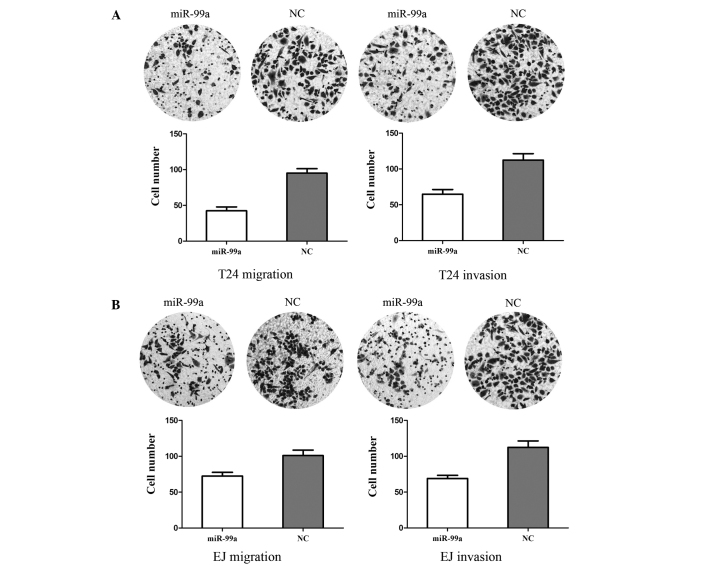
miR-99a inhibits cell migration and invasion in bladder cancer cell lines. Subsequent to a 12-h incubation, the number of (A) T24 and (B) EJ cells that transversed the Transwell membrane was significantly decreased after transfection of miR-99a (Student’s t-test, P<0.05). Subsequent to a 24-h incubation, the number of T24 and EJ cells that transversed the Transwell membrane precoated with Matrigel significantly decreased following transfection with miR-99a (Student’s t-test, P<0.05). miRNA-99a, microRNA-99a; NC, scrambled control.

In order to measure the effect of miR-99a on tumor cell migration, the Transwell apparatus assay was used (Fig. 2). In the migration assay, miR-99a-transfected cells exhibited a 49.54±7.46% decrease in T24 cells and a 31.32±6.69% decrease in EJ cells, compared with the NC-transfected cells. In the invasion assay, miR-99a-transfected cells demonstrated a 42.34±5.89% decrease in T24 cells and a 38.62±7.85% decrease in EJ cells, compared with the NC-transfected cells. These results indicated that miR-99a reduced the migration and invasion in bladder cancer T24 and EJ cells.

### FGFR3 is downregulated following overexpression of miR-99a in T24 and EJ cells

**Figure 3:**
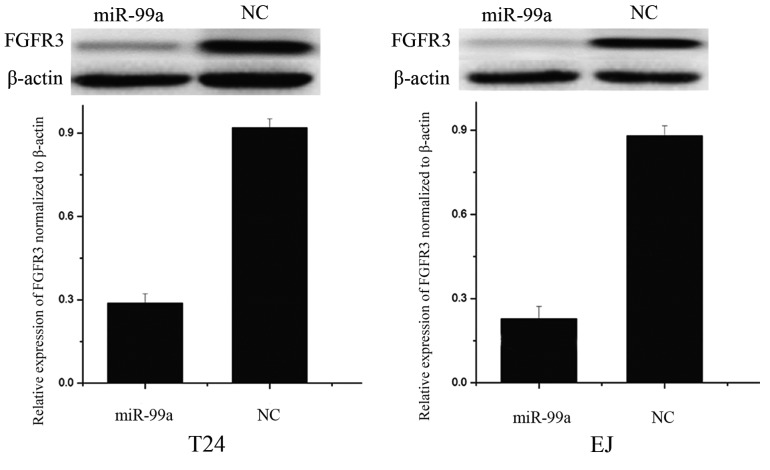
FGFR3 expression was significantly decreased in T24 and EJ cells after transfection of miR-99a. FGFR3, fibroblast growth factor receptor 3; miR-99a, microRNA-99a. Student’s t test, P<0.05

Western blot analysis was performed to analyze whether FGFR3 was decreased following transfection of the miR-99a mimics in the bladder cancer cell lines, T24 and EJ. As shown in Fig. 3, FGFR3 was significantly downregulated in the bladder cancer T24 and EJ cell lines subsequent to overexpression of miR-99a (P<0.05). These results indicated that miR-99a reduced the protein level of FGFR3 in the bladder cancer cells.

### FGFR3 is a direct target gene of miR-99a in bladder cancer

**Figure 4:**
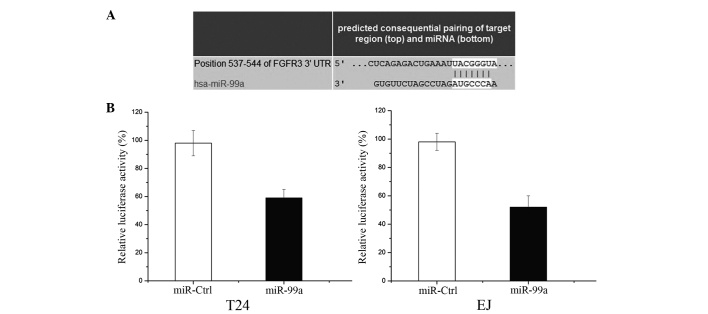
(A) TargetScan assessed that the FGFR3 mRNA contained a miR-99a seven-nucleotide seed match at position 537–544 of the FGFR3 3′-UTR. (B) FGFR3 may be a direct target of miR-99a *in vitro*. The luciferase activity was significantly lowered when cotransfected with miR-99a and a reporter plasmid in T24 and EJ cells. The overexpression of miR-99a could suppress the FGFR3 3′UTR-luciferase activity by 39% in T24 and 46% in EJ cells. FGFR3, fibroblast growth factor receptor 3; UTR, untranslated region; miR-99a, microRNA-99a. Student’s t test, P<0.05

In order to determine whether miR-99a targets the FGFR3 3′-UTR, TargetScan 5.2 and PicTar were used to assess the complementarity of miR-99a to the FGFR3 3′-UTR. It was shown that FGFR3 mRNA contained an miR-99a seven-nucleotide seed match at position 537–544 of the FGFR3 3′-UTR (Fig. 4A).

Luciferase reporter assays were performed to evaluate whether FGFR3 is a bona fide target of miR-99a. As shown in Fig. 4B, overexpression of miR-99a could suppress the FGFR3 3′UTR-luciferase activity by 39% in T24 cells and 46% in EJ cells (P<0.05). Above all, FGFR3 may be a direct target of miR-99a *in vitro*.

## Discussion

It has been verified that miRNAs regulate the expression of approximately one-third of the human genes. Thus, dysregulation of miRNAs is expected in human diseases, including cancer, which is attributed to dysregulated gene expression of tumor suppressor and oncogenes. Investigation of the differentially expressed miRNAs in cancer specimens has yielded significant information on carcinogenesis. Although bladder cancer generally carries a favorable prognosis, the five-year survival rate for localized disease is only 47% for regional cancers and 6% in those with distant metastasis (12). Therefore, it is important to elucidate the molecular pathways involved in bladder cancer carcinogenesis in order to improve the diagnosis and therapy of the disease.

It has been reported that miR-99a is transcribed from the commonly deleted region at 21q21 in human lung cancers (13). Expression of miR-99a has been indicated to be frequently downregulated in various tumors, including squamous cell carcinoma of the tongue, lung cancer, renal cell carcinoma (RCC), HCC, ovarian carcinoma, bladder cancer, childhood adrenocortical tumors and prostate cancer (14). In RCC, it was demonstrated that deregulation of miR-99a is involved in the etiology of RCC partially via direct targeting of the mammalian target of rapamycin (mTOR) pathway. It was also shown that decreased miR-99a expression in RCC clinical samples correlates with the overall survival rate of RCC patients and the suppression of tumorigenicity upon upregulation of miR-99a *in vitro* and *in vivo* (11). In HCC, miR-99a expression has been found to correlate with HCC patients’ survival rate, and miR-99a restoration suppresses HCC growth by targeting insulin-like growth factor 1 and mTOR (14). Therefore, upregulating miR-99a or providing analogous pharmaceutical compounds exogenously should be effective cancer therapies for tumors resulting from the activation or overexpression of these oncogenes.

In the present study, it was identified that miR-99a may function as a tumor suppressor through repression of FGFR3 in bladder cancer. miR-99a transfection resulted in decreased cell viability, and reduced migration and invasion in bladder cancer cells. Our findings indicated that miR-99a may have a potential therapeutic role by regulating oncogenes in bladder cancer patients.

FGFR3 is implicated as an oncogene in the majority (~80%) of low-grade non-invasive (stage Ta) bladder tumors and ~40% of invasive bladder tumors (15). FGFR3 belongs to a family of structurally related tyrosine kinase receptors (FGFR1-4), which are involved in numerous aspects of various biological processes, including proliferation, differentiation, migration and apoptosis (16,17). FGFR3 is composed of three immunoglobulin-like domains, a single hydrophobic membrane-spanning segment and a cytoplasmic tyrosine kinase domain (18). It is activated by an FGF ligand binding to the extracellular immunoglobulin-like domains II and III. Subsequently, trans-autophosphorylation at the tyrosine residues in the cytoplasmic domain is required for the stimulation of the intrinsic catalytic activity and activation of downstream signaling pathways. Activation of FGFR3 is thought to result in the stimulation of the Ras/Raf/MEK/ERK pathway (also known as the mitogen activated protein kinase pathway) and the PI3K/Akt pathway in bladder cancer (19). It has been shown to mediate growth and neoplasia in colorectal (20), bladder (21) and oral (22) cancers.

The activation of FGFR3 typically occurs through mutations within the extracellular and transmembrane domains and overexpression of wild-type receptor (23). FGFR3 mutations are found in >70% of low-grade, noninvasive bladder cancers and in 15–20% of high-grade tumors (19). Several studies have established a causal link between the presence of FGFR3-activating mutations and tumorigenesis. Tomlinson *et al* (23) stably knocked down mutant FGFR3 in the 97-7 S249C mutant urothelial cell line, which resulted in attenuation of cell growth and colony formation. Overexpression of the S249C mutant in 97-7 cells restored these neoplastic features, establishing the role of overactive FGFR3 as the tumorigenic driver within this cell line (19).

The overexpression of FGFR3 has also been observed in a number of tumor panels in bladder cancer (24–26). Immunohistochemical staining for FGFR3 in a panel of 126 bladder cancer tumors revealed intense staining in 20 (15.9%) samples and moderate staining in 42 (33.3%) samples (24). In a second study, FGFR3 staining of a tissue microarray comprising 257 pTa and pT1 bladder cancer samples revealed a significant association between FGFR3 overexpression and well-differentiated, early stage tumors (25). Gómez-Román *et al* found a correlation between FGFR3 overexpression at the transcript and protein level in low-grade, non-muscle invasive tumors (26). Tomlinson *et al* first correlated the FGFR3 mutation status with overexpression in a panel of 158 bladder cancer tumors (27). Therefore, FGFR3 deserves close scrutiny as a potential target for the inhibition in bladder cancer. The results of the present study indicated that miR-99a suppressed bladder cancer cell proliferation, migration and invasion by the downregulation of FGFR3. These could be investigated as targets of therapeutic drugs for bladder cancer.

In summary, to the best of our knowledge, the present study was the first to show that miR-99a regulates FGFR3 and contributes to cell proliferation, migration and invasion in bladder cancer. The identification of candidate target genes of miR-99a may provide an understanding of potential carcinogenic mechanisms in bladder cancer. These findings have therapeutic implications and may be exploited for further treatment of bladder cancer.

Future studies are required to address whether the potential of miR-99a may be fully realized in cancer treatment. If so, miRNA-99a may be beneficial for the treatment of bladder cancer.
